# Hypovolemia explains the reduced stroke volume at altitude

**DOI:** 10.1002/phy2.94

**Published:** 2013-10-02

**Authors:** Christoph Siebenmann, Mike Hug, Stefanie Keiser, Andrea Müller, Johannes van Lieshout, Peter Rasmussen, Carsten Lundby

**Affiliations:** 1 Center for Integrative Human Physiology, Institute of Physiology, University of ZurichZurich, Switzerland; 2 Intensive Care Unit, Department of Internal Medicine, University HospitalZurich, Switzerland; 3 Laboratory for Clinical Cardiovascular Physiology, Department of Internal Medicine, Academic Medical Centre F7N-252, University of AmsterdamAmsterdam, the Netherlands; 4 MRC/Arthritis Research UK Centre for Musculoskeletal Ageing Research, School of Biomedical Sciences, Queen's Medical Centre, University of Nottingham Medical SchoolNottingham, U.K

**Keywords:** Acclimatization, blood, Frank–Starling, heart, hypoxia

## Abstract

During acute altitude exposure tachycardia increases cardiac output (Q) thus preserving systemic O_2_ delivery. Within days of acclimatization, however, Q normalizes following an unexplained reduction in stroke volume (SV). To investigate whether the altitude-mediated reduction in plasma volume (PV) and hence central blood volume (CBV) is the underlying mechanism we increased/decreased CBV by means of passive whole body head-down (HDT) and head-up (HUT) tilting in seven lowlanders at sea level (SL) and after 25/26 days of residence at 3454 m. Prior to the experiment on day 26, PV was normalized by infusions of a PV expander. Cardiovascular responses to whole body tilting were monitored by pulse contour analysis. After 25/26 days at 3454 m PV and blood volume decreased by 9 ± 4% and 6 ± 2%, respectively (*P* < 0.001 for both). SV was reduced compared to SL for each HUT angle (*P* < 0.0005). However, the expected increase in SV from HUT to HDT persisted and ended in the same plateau as at SL, albeit this was shifted 18 ± 20° toward HDT (*P* = 0.019). PV expansion restored SV to SL during HUT and to an ∼8% higher level during HDT (*P* = 0.003). The parallel increase in SV from HUT to HDT at altitude and SL to a similar plateau demonstrates an unchanged dependence of SV on CBV, indicating that the reduced SV during HUT was related to an attenuated CBV for a given tilt angle. Restoration of SV by PV expansion rules out a significant contribution of other mechanisms, supporting that resting SV at altitude becomes reduced due to a hypovolemia.

## Introduction

Exposure to high altitude reduces arterial O_2_ content which, by stimulation of peripheral chemoreceptor activity, triggers an increase in cardiac output (Q) that preserves systemic O_2_ delivery (Downing et al. 1962). The rise in Q is mainly the consequence of an accelerated heart rate (HR) whereas cardiac stroke volume (SV) is initially barely affected (Naeije 2010). However, although tachycardia persists throughout altitude acclimatization, Q returns to or even below sea level (SL) values within a few days due to a decrease in SV (Vogel and Harris 1967). The physiological mechanism for this is incompletely understood.

Left ventricular (LV) ejection fraction is unaffected or even slightly enhanced in hypoxia (Suarez et al. 1987; Hirata et al. 1991), suggesting that the lower SV is the consequence of a diminished LV end-diastolic volume. The latter has repeatedly been observed (Alexander and Grover 1983; Fowles and Hultgren 1983; Suarez et al. 1987; Hirata et al. 1991) and may at least partially be associated with an adverse influence of hypoxia on diastolic LV function (Gomez and Mink 1992; Kullmer et al. 1995). Another underlying mechanism may relate to the vasoconstrictive response of the pulmonary circulation to alveolar hypoxia (Moudgil et al. 2005). The subsequent increase in afterload may attenuate right ventricular SV (Naeije and Rondelet 2004) while the pressure overload may shift the interventricular septum towards the LV cavity (Gan et al. 2006) and induce pericardial constraint (Fujimoto et al. 2011), all compromising LV diastolic filling volume. Nevertheless, changes in arterial oxygenation and pulmonary artery pressure commence immediately after the onset of altitude exposure and revert rapidly after return to SL (Moudgil et al. 2005) whereas the decrease in SV only occurs after a few days and persists for 1–2 days after return to SL (Reeves et al. 1987). Therefore, the reduction in end-diastolic LV volume at altitude may rather relate to slower occurring hematological changes.

After a few days of acclimatization to high altitude a reduced plasma volume (PV) is a universal finding (Hannon et al. 1969a,b; Rasmussen et al. 2013). This increases arterial hemoglobin concentration ([Hb]) (Singh et al. 1990) but also induces a reduction in total blood volume that may attenuate diastolic filling by diminishing central blood volume (CBV).

In this study, we examined the contribution of the altitude-related decrease in PV to the reduced SV by means of passive whole body tilting and PV expansion. During postural changes gravity affects the distribution of venous blood so that CBV decreases with head-up tilt (HUT) and increases with head-down tilt (HDT) (Harms et al. 2003; Truijen et al. 2010). In response, SV progressively increases from HUT to HDT. However, in normovolemic subjects SV plateaus in the supine position and remains unaffected by HDT, indicating that the flat portion of the Frank–Starling curve is reached (Harms et al. 2003; van Lieshout et al. 2005). If hypovolemia reduces CBV and thus SV at altitude, the transition from HUT to HDT should evoke the same increase in both but with lower values for a given tilt angle. Furthermore, although SV should eventually reach the same plateau, this should be shifted toward HDT level (Truijen et al. 2010). We therefore compared the cardiovascular response of lowlanders to HUT and HDT at SL, after 25 days of acclimatization to 3454 m, and again the following day after restoration of blood volume to SL values by PV expansion. We hypothesized that at altitude (1) SV would be reduced for a given HUT angle but reveal an increase toward the supine position similar to that at SL, (2) SV reaches the same plateau as at SL but this would be shifted toward HDT, and (3) restoration of blood volume would normalize the SV response to whole body tilting.

## Methods

This study was conducted at the University of Zurich (∼500 m, referred to as SL) and the Jungfraujoch research station (3454 m) in the Swiss Alps and was approved by the ethical committee of the Swiss Federal Institute of Technology (EK 2011-N-51) and conducted in accordance with the Declaration of Helsinki.

### Subjects

Seven healthy, male, Caucasian lowlanders (26 ± 4 years; 180 ± 1 cm; 76 ± 6 kg) were recruited as study participants and gave oral and written consent. All were physically active but not involved in elite sport. To avoid influence from previous hypoxic exposure persons that had traveled to altitudes >2000 m within the last 4 weeks before the onset of the study were excluded.

### Overall protocol

The baseline study period at SL lasted 5 weeks and included four measurements of intravascular volumes by carbon monoxide (CO) rebreathing, all of them separated by 7–12 days, assessment of resting pulmonary artery pressure by Doppler echocardiography and the first tilt table experiment.

At the onset of the altitude period subjects were transported by train to the Jungfraujoch research station which offers bedrooms, kitchen facilities, and living space with normal room temperatures. The sojourn lasted 4 weeks over which the subjects did not descend below the altitude of the station. They maintained their usual physical activity by hiking, mountaineering activities, and exercising on a cycle ergometer. Subjects were encouraged to pursue their habitual diet for which they ordered their desired groceries. During the altitude period, duplicate measurements of intravascular volumes were performed after 2 and 3 weeks and Doppler assessments of pulmonary artery pressure after 3 weeks. The tilt table experiments were repeated after 25 and again after 26 days at altitude. Prior to the last tilt table experiment blood volume was restored to baseline levels by intravenous infusion of isotonic Dextran solution (Voluven, Fresenius Kabi, Bad Homburg, Germany).

After the altitude period subjects were transported back to Zurich by train.

### Intravascular volumes

Intravascular volumes were determined by a modified version of the CO rebreathing protocol of Burge and Skinner (1995). After transcutaneous insertion of an 18 gauge catheter into an antecubital vein subjects rested for 20 min in a semi-recumbent position. During this period they drank 0.5 L of water to preclude dehydration. Their legs were elevated to facilitate venous return from the lower extremities.

Subjects then breathed pure O_2_ through an open circuit from a Douglas bag for 4 min to eliminate N_2_ from the airways. Thereafter, the breathing circuit (previously O_2_ flushed) was closed by a sliding valve. After a few breaths a priming dose (20 mL) of 99.997% chemically pure CO (N47, Air Liquide, Pullach, Germany) was administrated as a bolus and rebreathed for 10 min while a soda lime container eliminated CO_2_ from the circuit. At the end of this period, 2 mL of venous blood was sampled and analyzed in quadruplicate for percent carboxy-hemoglobin (%HbCO) and [Hb] in a hemoximeter (ABL800, Radiometer, Copenhagen, Denmark). Furthermore, hematocrit was determined by the micromethod (4 min at 13,500 rpm). While the subjects remained connected, the rebreathing circuit was then flushed with pure O_2_ and the main CO dose was administered (1.2 mL/kg bodyweight) and rebreathed for 10 min. Thereafter, a second 2 mL blood sample was obtained and analyzed. The change in %HbCO between the first and second blood sample (ΔHbCO) was used for calculation of total hemoglobin mass (= 0.978 × nCO × 25/ΔHbCO, with nCO being the number of CO molecules in the second dose), taking into account the ∼2.2% of CO remaining in the rebreathing circuit at the end of the rebreathing period (Burge and Skinner 1995). Red blood cell volume (= hemoglobin mass × hematocrit/[Hb]), blood volume (= red cell volume × 100/hematocrit), and PV (= blood volume – red cell volume) were then calculated (Burge and Skinner 1995).

To account for the reduced barometric pressure the CO doses were increased to 30 mL (priming dose) and 1.5 mL/kg (main dose) during the altitude period. All CO rebreathing tests were performed by the same operator as duplicate measurements on subsequent days. The typical measurement errors (SD of difference scores/√2) for blood volume, red cell volume and PV, assessed from the first duplicate measurement at SL, were 2.6%, 1.6%, and 3.3%, respectively.

The here reported SL values for intravascular volumes correspond to the average over the four duplicate measurements conducted during the baseline period. Similarly, the intravascular volumes at altitude correspond to the average over the duplicate measurements conducted after 2 and 3 weeks.

### Tilt table experiments

Subjects rested in the supine position on the tilt table with their feet strapped into a retainer. A fixed bicycle saddle was adjusted to support their body weight during HUT. After 5 min in the horizontal position the subjects were tilted to −90°, −60°, and −30° (i.e., HDT) and to 15°, 30°, and 60° (i.e., HUT), respectively, remaining in each position for 3 min. The order of the positions was randomized but HUT was always alternated with HDT, and vice versa.

Throughout the tilt table experiments mean finger arterial pressure was measured by the volume clamp method as the integral over one heart beat (Finometer PRO; Finapres Medical Systems B.V., Amsterdam, the Netherlands). HR was the inverse of the inter beat interval. SV was determined by a three-element model of arterial input impedance (Modelflow, Finometer PRO, Finapres Medical Systems B.V., Amsterdam, the Netherlands) incorporating age, sex, height, and weight from the blood pressure waveform (Wesseling et al. 1993). Data were recorded at a frequency of 1 kHz (Powerlab; ADInstruments, Bella Vista, Australia). For the analysis, the measurements over the last 30 sec in each tilt position were averaged.

### PV expansion

Prior to the second tilt table experiment at altitude subjects' blood volumes were restored to baseline by infusion of isotonic Dextran solution. The difference between blood volume at SL and at altitude was supplemented through a peripheral venous catheter with the subjects blinded toward the volume. The subsequent tilt table experiment was initiated immediately following completion of the PV expansion.

### Pulmonary artery pressure

Transthoracic echocardiography (CX50 Ultrasound system, Philips, Amsterdam, the Netherlands) was performed in the supine position at SL and after 3 weeks at altitude. Systolic pulmonary artery pressure was estimated from the peak tricuspid regurgitation jet velocity, using the simplified Bernoulli equation and combining this value with an estimate of the right atrial pressure. Right atrial pressure was estimated from the inferior vena cava diameter and respiratory changes (Rudski et al. 2010). The reported results conform to averages over three individual heart beats.

### Blood pressure

In addition to the blood pressure measurements performed during the tilt table experiments, oscillometric arterial blood pressure was measured in triplicate (Dinamap Pro Care 100, GE Medical Systems Information Technologies GmbH, Freiburg, Germany) prior to each assessment of intravascular volumes, with the subjects supine. The here reported SL values correspond to the average over all measurements conducted during the baseline period whereas the altitude values correspond to the average over the measurements conducted prior the CO rebreathings performed after 2 and 3 weeks at altitude.

### Statistics

The hemodynamic responses to the tilt table experiments at the different time points were compared by application of a general linear model (GLM) with Tukey–Kramer post hoc test. For the analysis the data were categorized as HUT, supine, and HDT.The tilt angle at which a plateau in SV was reached over the range from HUT to HDT was estimated by a two-segment linear regression analysis that determined the tilt angle at which the breakpoint between the two regression lines was located.Changes in intravascular volumes, blood pressure, and pulmonary artery pressure from SL to altitude were evaluated by paired *t*-tests. Statistics were performed using SAS 9.3. (SAS Institute Inc., Cary, NC) and Sigmaplot 11.0 (Systate software, Chicago, IL). A *P*-value <0.05 was considered as statistically significant.

## Results

### Blood withdrawal over the study

During the baseline period weekly blood withdrawals for the experiments reported here and for other study purposes ranged from 4–50 mL. At altitude another 10–65 mL/week were withdrawn. The total volume of blood withdrawal was ∼150 mL over the baseline period and ∼180 mL over the altitude period.

### Hematological changes

The four duplicate CO rebreathings at SL revealed no significant changes in blood volume and red cell volume whereas PV was increased by 6% 2 weeks before the onset of the altitude period (*P* = 0.006 vs. first measurement) but had normalized 1 week later. At altitude, no differences in either blood volume, PV, or red cell volume were observed between the measurements conducted after 2 and 3 weeks. The results obtained at SL and altitude were then averaged for comparison (Table 1). At altitude, PV and blood volume were reduced by 9 and 6%, respectively (*P* < 0.001 for both) while red cell volume remained unchanged. As a result, [Hb] and hematocrit both increased at altitude.

**Table 1:** Hematological variables at sea level and after 3 weeks at 3454 m altitude

	Sea level	Altitude	*P*-value
Blood volume (L)	6.03 ± 0.28	5.69 ± 0.27	<0.001
Plasma volume (L)	3.44 ± 0.24	3.11 ± 0.22	<0.001
Red cell volume (L)	2.59 ± 0.18	2.58 ± 0.24	0.78
Hematocrit (%)	42.9 ± 2.6	45.3 ± 3.2	0.008
[Hb] (g/L)	145 ± 10	154 ± 10	0.002

[Hb], venous hemoglobin concentration.

The volume of isotonic Dextran solution required to restore blood volume at altitude was 339 ± 143 mL.

### Stroke volume

**Figure 1:**
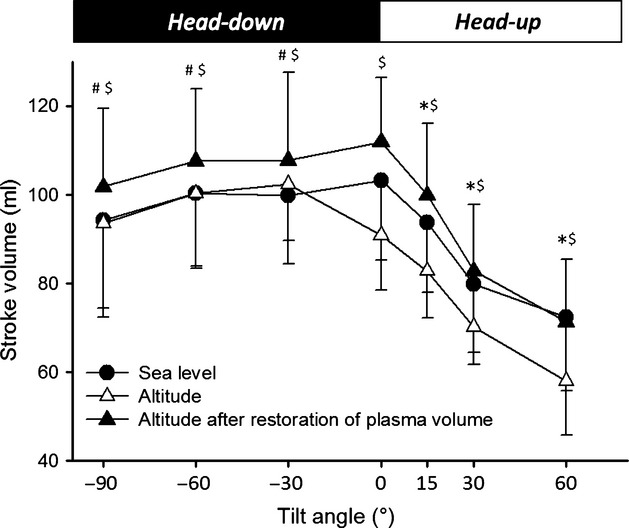
The stroke volume (SV) response (mean +/− SD) to whole body tilting at sea level (SL), after 25 days at 3454 m altitude, and on the subsequent day after restoration of plasma volume. SV increased from head-up tilt to supine (*P* < 0.0001) whereas during head-down tilt it was independent of the tilt angle. *P < 0.05 for altitude versus SL; ^#^*P* < 0.05 for altitude after hemodilution versus SL; ^$^*P* < 0.05 for altitude versus altitude after hemodilution.

In all tilt table experiments, we observed a similar increase in SV from HUT to HDT (*P* < 0.0001) that led to a plateau where after SV remained independent of the tilt angle (Fig. 1). Nevertheless, SV was lower at altitude than at SL for a given HUT angle (*P* < 0.0005) but not in the supine position (*P* = 0.3) or during HDT (*P* = 0.7). Two-segment linear regression indicated that, at SL, the onset of the SV plateau was located at a tilt angle of 0.8 ± 9° (*P* = 0.9 vs. supine position) whereas, at altitude, it was shifted 18 ± 20° toward HDT (*P* = 0.019).

Normalization of blood volume increased SV in all tilt positions (HDT, *P* = 0.003; supine position, *P* = 0.04; HUT, *P* < 0.0001) (Fig. 1). As a consequence, SV was similar to baseline during HUT and in the supine position but ∼8% higher during HDT (*P* = 0.03). The onset of the SV plateau had reverted to a tilt angle that was not significantly different from the supine position (−5 ± 9°, *P* = 0.5).

### HR, Q, and systemic blood pressure

At altitude HR was accelerated compared to SL in all tilt positions. While HUT always induced an increase in HR, this was steeper at altitude (prior to PV expansion) than at SL (*P* = 0.04). The PV expansion did not affect HR during HDT but flattened the HR response to HUT (*P* = 0.04) (Table 2).

**Table 2:** Cardiovascular responses to whole body tilting at sea level, after 25 days at 3454 m altitude, and on the subsequent day after restoration of plasma volume

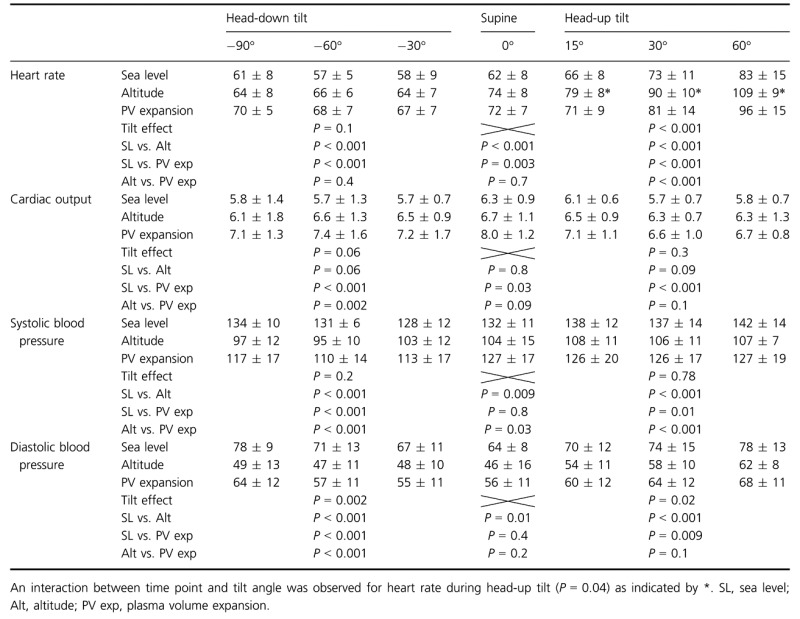

Cardiac output always remained unaffected by changes in tilt position. At altitude, Q was similar to SL whereas PV expansion increased Q throughout all tilt angles.

According to the volume clamp method both systolic and diastolic blood pressures were reduced at altitude and partially restored by the PV expansion. In all conditions systolic blood pressure remained unaffected by changes in tilt angle whereas diastolic blood pressure tended to be lowest in the supine position and increase toward both HUT and HDT. In contrast, oscillometry indicated a slight increase in blood pressure from SL to altitude with systolic and diastolic pressures raising from 126 ± 7 to 132 ± 6 mmHg and from 68 ± 6 to 73 ± 6 mmHg, respectively (*P* = 0.047 and *P* = 0.03).

### Systolic pulmonary artery pressure

Systolic pulmonary artery pressure was 17.4 ± 4.3 mmHg at SL. After 3 weeks of altitude exposure it had increased to 25.2 ± 5.8 mmHg (*P* = 0.005).

## Discussion

We investigated the contribution of hypovolemia to the reduction in SV at altitude. After 3 weeks at 3454 m blood volume had decreased by 6% due to a 9% PV contraction. SV during HUT was lower than at SL but the increase toward the supine position, and the plateau that was eventually reached, were similar. At SL the onset of the SV plateau was located in the supine position whereas it was shifted 18 ± 20° toward HDT at altitude (*P* = 0.019). Normalization of PV at altitude restored SV to SL during HUT and to ∼8% higher levels during HDT.

Hypoxia is omnipresent in modern human life with millions of people dwelling at or traveling to altitudes >2500 m as well as a major expression of cardiovascular and pulmonary diseases and its effects on organ function is thus of continued interest in medicine and physiology. In the early adaptation to altitude the initial increase in Q plays a key role as it preserves systemic O_2_ delivery despite arterial hypoxemia (Bartsch and Saltin 2008). Accordingly, the subsequent reduction in SV, that normalizes Q before complete restoration of arterial O_2_ content has been established (Naeije 2010), is intriguing. As SV is determined by myocardial contractility, cardiac preload and afterload (von Spiegel et al. 1998), each of these could mediate the negative inotropic effect of hypoxia. However, LV ejection fraction for a given end-diastolic volume is unaffected or even increased at altitude (Suarez et al. 1987; Hirata et al. 1991) which makes an adverse effect of hypoxia on myocardial contractility unlikely. The finding in this study that SV reached the same plateau as at SL when an adequate CBV was provided by HDT are in line with this notion. Likewise, altitude-induced changes in LV afterload are unlikely to reduce SV as the local vasodilatory responses to hypoxia counteract the systemic vasoconstrictive effect of an increased sympathetic nervous activity (Blitzer et al. 1996). Accordingly, the reduced SV seems to be related to changes in LV preload in line with studies reporting a lower end-diastolic volume at altitude (Alexander and Grover 1983; Fowles and Hultgren 1983; Suarez et al. 1987; Hirata et al. 1991). The contraction of PV that develops within the first days of exposure to altitude (Singh et al. 1990) may affect diastolic filling by attenuating CBV (Kirwan et al. 1981). Experimental support for this was provided by Grover and colleagues who demonstrated that inspiratory CO_2_ supplementation during hypoxic exposure prevents both the PV contraction and the reduction in SV (Grover et al. 1976). The intervention, however, also prevented the normally occurring hypoxia induced hypocapnia (Siebenmann et al. 2013). As hypocapnia may itself impair diastolic function (Bing et al. 1973; Gomez and Mink 1992), the CO_2_ supplementation might have preserved SV independently from the maintained PV. To avoid this potential limitation we manipulated CBV by whole body tilting where the transition from HUT to HDT facilitates venous return from large vessels in the lower extremities (Harms et al. 2003; Truijen et al. 2010). The resulting increase in CBV augments the ventricular end-diastolic volumes and thus SV by the Frank–Starling mechanism (Harms et al. 2003; Truijen et al. 2010). However, in normovolemic individuals SV reaches a plateau in the supine position with no further increase during HDT, indicating that the Frank–Starling mechanism is fully exploited (Harms et al. 2003; Truijen et al. 2010) and this was also confirmed in our subjects at SL. At altitude both the increase in SV from HUT to HDT and the plateau for SV that was eventually reached appeared similar to what was observed at SL, revealing that the dependence of SV on CBV was unchanged. However, the SV response curve was shifted so that during HUT a given SV was reached in a tilt position that was closer to supine and, similarly, slight HDT was required to evoke a plateau. Together these findings indicate that SV during HUT was reduced due to a lower CBV for a given tilt angle. During HDT at altitude CBV was presumably still lower than at SL but as it was sufficient to fully exploit the Frank–Starling mechanism, this did not result in a reduced SV.

The PV expansions allowed determining whether CBV was attenuated due the 6% reduction in blood volume or other mechanisms. As the PV expansion restored SV to or even beyond baseline levels we conclude that hypovolemia accounted for the entire reduction in CBV and thus SV. However, as HDT evoked a plateau in SV already before hemodilution, the ∼8% increase in SV after hemodilution was likely independent of changes in CBV. Hemodilution may have increased SV further by enhancing myocardial contractility (Lew 1993) and/or reducing LV afterload by lowering circulating catecholamines (Calvin et al. 1981; Kumar et al. 2004a,b), stimulating low pressure baroreceptors (Oberg and Thoren 1972), and promoting atrial natriuretic peptide release (Legault et al. 1992). During HUT these mechanisms were probably counteracted by the baroreflex-induced vasoconstriction and tachycardia.

Besides hypovolemia, hypoxic pulmonary vasoconstriction is another mechanism that may, under given circumstances, attenuate SV at altitude (Moudgil et al. 2005). In normoxia hypoxic pulmonary vasoconstriction optimizes the matching of ventilation and perfusion without affecting pulmonary artery pressure, but in hypoxia pulmonary vasoconstriction leads to a rapid rise in pulmonary artery pressure (Naeije 1992, 1997). The consequent increase in afterload may attenuate right ventricular SV and, in turn, LV end-diastolic volume (Nootens et al. 1995; Naeije and Rondelet 2004). In addition, pressure overload may dilate the right ventricle and further compromise LV end-diastolic volume by inducing interventricular septum shift (Badke 1982; Gan et al. 2006) and pericardial constraint (Fujimoto et al. 2011). In previous studies pharmacological attenuation of hypoxic pulmonary vasoconstriction has indeed elevated SV during exercise at altitude (Ghofrani et al. 2004; Hsu et al. 2006). Nevertheless, despite a ∼45% increase in systolic pulmonary artery pressure SV at altitude was fully restored in our subjects following PV expansion. Moreover, although HDT may further aggravate pulmonary artery pressure (van Lieshout et al. 2005) HDT evoked a similar SV plateau at altitude as at SL. Together these findings indicate that, in normal individuals, the hypoxic pulmonary vasoconstriction does not impair SV at altitude during resting conditions. A restricting effect may, however, occur when the circulatory demand and/or pulmonary artery pressure are further elevated by exercise (Ghofrani et al. 2004; Hsu et al. 2006) although this has been challenged in other studies (Ricart et al. 2005; Bernheim et al. 2007).

Besides revealing the mechanism that reduces SV at altitude, this study illustrated the ability of the baroreflex to compensate for acute changes in SV by adjusting HR and thus maintain Q within a narrow range (Westerhof et al. 2006; Fu et al. 2012). As SV during HUT at altitude was not only attenuated by orthostatic stress, but also by hypovolemia, the baroreflex may have been activated more thus explaining the steeper increase in HR. This is further suggested by the PV expansion which flattened the increase in HR during HUT. These observations enhance our understanding of the mechanisms that maintain tachycardia throughout altitude exposure, as they suggest a contribution of the baroreflex at least in the upright position.

We have chosen a noninvasive technique to quantify SV as invasive procedures may among others affect vascular tone and thus CBV. Although echocardiography may have allowed to estimate end-diastolic and end-systolic volumes this technique may be flawed during passive whole body tilting due to internal organ shifts (van Lieshout et al. 2003). Furthermore, as the acute hemodynamic response to orthostatic stress is followed by slower changes in SV (van Lieshout et al. 2005) it was a necessity to avoid variations in measurement time and for this purpose the pulse contour analysis was superior. Although this method might be affected by changes in vascular compliance, a previous validation has demonstrated that the Modelflow determined SV/Q correctly tracks SV/Q as determined by thermodilution despite the progressive increase in vascular tone induced by severe orthostatic stress as induced by prolonged HUT for 1 h (Harms et al. 1999). As the cardiovascular responses to orthostatic stress and hypoxia are similar we expected the pulse contour analysis to be valid also at altitude.

We also used the Finometer to monitor systemic blood pressures during the tilt table experiments. This revealed a parallel response to the tilting protocol in all conditions, however, contrary to oscillometry and to common expectations a lower blood pressure at altitude than at SL. According to the manufacturers this presumed underestimation may have been related to a suboptimal performance of the integrated pump. The built-in dynamic servo setpoint adjuster (Physiocal) defines and maintains the diameter at which the finger artery is clamped, keeping transmural vascular pressure across the finger arterial wall constant during the measurement. As long as constancy of transmural vascular pressure is maintained, environmental barometric pressure does not affect vascular compliance. In this regard it is important to note that, in a previous validation of Modeflow determination of SV/Q, simulation of a systematic offset in blood pressure over a range of 20 mmHg did only minimally affect the measurement of SV/Q (Wesseling et al. 1993).

Over the course of the study we repeatedly collected blood for experimental purposes. At altitude the blood withdrawal conformed to ∼3% of the initial red cell volume and may have offset the 2.5–5% increase in red cell volume that can be expected to occur during 3 weeks at ∼3500 m (Rasmussen et al. 2013). It should be considered whether this may have affected the total blood volume and thus the outcome of the tilt table experiments. Pottgiesser et al. 2008 demonstrated red cell volume recovery within 5 weeks following a 550 mL whole blood donation. After a shortly delayed onset the daily recovery rate corresponded to 2.1 g of hemoglobin, that is, ∼6 mL of red cell volume. In this study, the weekly loss in red cell volume due to blood sampling was 2–30 mL. We therefore assume that, at least during the baseline period, the red cell volume loss was continuously recovered, as suggested by the four measurements that revealed no significant changes. As indicated, however, we cannot exclude that the blood withdrawal prevented a slight red cell volume expansion at altitude. Nevertheless, blood volume recovers rapidly after phlebotomy as PV increases above initial levels to compensate for the loss in red cell volume (Pottgiesser et al. 2008). We therefore argue that the effect of blood sampling on total blood volume and thus cardiac preload was probably neglectable.

In conclusion our results indicate that a lower blood volume is the mechanism responsible for the reduced resting SV at 3454 m altitude whereas myocardial contractility is preserved. The complete restoration of SV to or above baseline levels after normalization of PV rules out contribution of mechanisms other than hypovolemia.
